# The geography of waiting: surveillance latency as the hidden driver of Ebola risk in East Africa

**DOI:** 10.3389/fpubh.2026.1899333

**Published:** 2026-10-06

**Authors:** Okechukwu Paul-Chima Ugwu, Chinyere Nneoma Ugwu, Godson Emeka Anyanwu, Mariam Basajja, Maryann Chiamaka Ebuoh

**Affiliations:** 1 Department of Research, Publication and Extension, Kampala International University, Kampala, Uganda; 2 Department of Anatomy, Faculty of Biomedical Sciences, Kampala International University, Kampala, Uganda; 3 Department of Human Anatomy, Enugu State University of Medical and Applied Sciences, Enugu, Nigeria

**Keywords:** early warning systems, East Africa, Ebola virus disease, epidemic preparedness, health security, outbreak detection, public health surveillance, surveillance latency

## Abstract

Ebola virus disease remains a persistent public health threat in East Africa, yet preparedness strategies continue to emphasise response after outbreaks are recognised rather than the period preceding detection. This Perspective argues that surveillance latency the cumulative delay between the earliest clinically meaningful signal and organised public health action is a critical but insufficiently measured determinant of Ebola risk. Although diagnostic and reporting delays have been widely documented, they are often treated as isolated operational challenges. We introduce the concept of the geography of waiting, an integrative framework that links biological, clinical, diagnostic, administrative and community delays to spatial inequalities in physical, technical and relational infrastructure. Unequal access to diagnostics, transportation, laboratory networks, trusted communication channels and community reporting systems creates substantial variation in detection timelines across settings. Where surveillance latency is prolonged, transmission may continue undetected, increasing the likelihood of larger and more complex outbreaks. Drawing on evidence from outbreaks in Uganda, the Democratic Republic of the Congo, Guinea and Rwanda, we examine the mechanisms underlying delayed detection and consider them alongside performance frameworks such as Uganda’s 7-1-7 strategy. We propose a practical approach that integrates syndromic and event-based surveillance, decentralised molecular testing, community early-warning systems and regional data sharing. Because shorter detection intervals are associated with smaller eventual outbreaks in the cases reviewed here, reducing surveillance latency is a plausible high-value investment for Ebola preparedness, although this remains a hypothesis pending formal economic evaluation rather than an established finding.

## Introduction

East Africa has become a critical region for Ebola preparedness. Uganda has experienced repeated Sudan virus disease outbreaks, while eastern Democratic Republic of the Congo has faced recurrent Ebola virus disease under conditions of conflict, population movement and strained health systems (1, 2). Clinical recognition remains difficult because early Ebola illness often resembles malaria, typhoid fever and other common febrile diseases (3–5). Preparedness has improved. The PALM trial changed treatment for Zaire ebolavirus disease by showing survival benefit with monoclonal antibody therapy (6). Ring vaccination with rVSV-ZEBOV has also become an important tool for Zaire ebolavirus outbreaks (7). Still, these advances do not solve late detection. A vaccine, treatment centre or response team cannot stop early transmission if the outbreak has not yet been recognised.

This Perspective shifts attention from response capacity to detection capacity. The central question is straightforward: how long does Ebola transmission continue before authorities know it exists? That interval is rarely foregrounded in preparedness metrics, yet it may determine whether a small cluster remains containable or becomes a regional emergency. The point is not that diagnostic or reporting delays are newly discovered; they are extensively documented, and performance frameworks such as the 7-1-7 target already measure time to detection, notification and early response (8). What has been missing is a way of treating these separate delays as one geographically distributed determinant rather than as isolated technical faults. To our knowledge, surveillance latency has not previously been conceptualised in this integrated, spatial form for Ebola preparedness in East Africa. We therefore introduce the geography of waiting as a framework that links outbreak detection delays with spatial inequalities in health-system access, diagnostic infrastructure and community reporting capacity, and that foregrounds the absence of enabling infrastructure as the underlying cause of waiting.

## Perspective scope and evidence base

This Perspective synthesises evidence from peer-reviewed literature, World Health Organization reports, Africa Centres for Disease Control and Prevention guidance documents, outbreak investigations and public health surveillance studies relevant to Ebola preparedness in East Africa. Sources were identified through searches of PubMed, Scopus, Web of Science and Google Scholar. The objective was not to conduct a systematic review but to develop a conceptual framework for understanding surveillance latency as a determinant of Ebola risk. Search terms included Ebola virus disease, Sudan virus disease, Bundibugyo virus, surveillance latency, outbreak detection, event-based surveillance, community-based surveillance, syndromic surveillance, diagnostic delay, 7-1-7 framework, Uganda Ebola, Democratic Republic of Congo Ebola, cross-border surveillance, health security and epidemic preparedness. Priority was given to peer-reviewed studies, WHO guidance, Africa CDC surveillance documents and outbreak investigations. Non-searchable or unverifiable sources were excluded.

## The geography of waiting: a conceptual framework

Preparedness is, in an important sense, a national characteristic. It is a core function of sovereign governance: a state assembles the laboratories, surveillance systems, workforce and response infrastructure needed to protect the health, safety and security of its citizens, and the capacity to detect threats and deploy countermeasures forms part of that sovereign responsibility. Yet national preparedness is not delivered uniformly across territory. The same sovereign system performs differently in a capital city than in a remote border district, because the infrastructure that converts a clinical signal into a public health action is not evenly distributed. A patient with suspected viral haemorrhagic fever at a referral hospital may be sampled quickly and linked to a national laboratory network. A similar patient in a peripheral district may wait days for clinical suspicion, specimen transport, testing, reporting and response activation. The geography of waiting describes this within-country, spatial inequality in detection time. It does not deny that preparedness is a national project; it draws attention to where that national project is thin on the ground.

This distinction matters because the cause of waiting in much of the global south is not principally a delayed decision by an alert official. More often it is the absence of the infrastructure that would allow timely action at all: the physical infrastructure of roads, electricity, cold chain and laboratories; the technical infrastructure of diagnostics, digital reporting and trained personnel; and the relational infrastructure of community trust and functioning escalation pathways. A health worker cannot send a specimen that no vehicle can carry, and a community cannot report through a channel it does not have or does not trust. The geography of waiting therefore treats latency as a symptom of missing infrastructure rather than as a failure of individual vigilance, and it locates the corrective task in building that infrastructure where it is absent. Framed this way, the concept does not assign blame to under-resourced districts; it makes their infrastructural deficits visible as measurable contributors to risk.

The framework aligns with event-based surveillance, which emphasises early collection, verification and interpretation of signals that may indicate acute public health risk (9, 10). It also reflects lessons from community-based surveillance during the 2018–2020 DRC outbreak, where local participation, rapid feedback and trusted communication were central to detection and response (11). Recent work on event-based surveillance monitoring and evaluation highlights the need for explicit indicators that measure how early warning systems detect, verify and act on signals across community, health-facility, border-point and media-based reporting channels (12). It is complementary to, rather than a replacement for, performance-target approaches such as the 7-1-7 framework: where 7-1-7 measures whether timeliness targets are met, the geography of waiting asks why they are missed in particular places and what infrastructure must be built to meet them.

## Situating surveillance latency among existing delay frameworks

Diagnostic and reporting delays have long been studied. The literature on viral haemorrhagic fever diagnostics describes the biosafety, logistical and laboratory-distribution constraints that slow confirmation (13), and outbreak investigations routinely document reporting bottlenecks. The most influential recent contribution is the 7-1-7 target, which sets explicit benchmarks of 7 days to detect an outbreak, 1 day to notify authorities and 7 days to complete early response actions (8). Uganda adopted 7-1-7 in 2021 and has applied it across a range of events. In a structured evaluation of 42 events presented between October 2021 and December 2022, 22 (52%) met the seven-day detection target and 31 (74%) met the one-day notification target, with bottleneck analysis used to identify why targets were missed (8). Subsequent applications to specific outbreaks, including a 2024 anthrax event in a south-western border district, have used the same metrics to pinpoint missed opportunities for early detection (14).

The geography of waiting is distinguished from these approaches in three ways. First, it is integrative rather than componentwise: instead of treating biological, clinical, diagnostic, administrative and community delays as separate problems, it binds them into a single cumulative interval whose length is the quantity of interest. Second, it is explicitly spatial: it treats latency as a property of places, predicting that the same national system will produce different detection times in different districts according to their infrastructure. Third, it is infrastructural in its causal account: where 7-1-7 measures whether a target was met, the geography of waiting asks which physical, technical or relational infrastructure was missing when it was not. The two are best seen as a measurement layer (7-1-7) and an explanatory and planning layer (geography of waiting) that together support decisions about where to invest. The framework thus extends rather than restates existing work on diagnostic and reporting delay.

Table 1 presents a conceptual framework of five surveillance latency domains that may delay Ebola outbreak detection in East Africa. For each domain, the principal source of delay, potential public health consequence, and corresponding corrective action are identified. The domains are interrelated and may overlap in practice, and each is shaped by the availability of physical, technical and relational infrastructure. The framework is intended to support preparedness planning, surveillance strengthening, and early outbreak detection.

**Table 1:** Surveillance latency domains and corrective actions for Ebola preparedness in East Africa.

Latency domain	Main source of delay	Public health consequence	Corrective action
Biological latency	Early symptoms resemble malaria, typhoid fever and other febrile illnesses	Ebola may not be suspected during the earliest infectious period	Strengthen clinical algorithms for severe or unexplained febrile illness
Clinical latency	Health workers initially prioritise common diagnoses	Suspected cases may be treated without isolation or notification	Train frontline staff in risk-based viral haemorrhagic fever triage
Diagnostic latency	Delays in specimen transport and laboratory confirmation; absence of decentralised testing infrastructure	Contact tracing and isolation begin late	Expand regional testing capacity, mobile laboratories and specimen transport systems
Administrative latency	Reporting bottlenecks and delayed escalation	Public health action lags behind laboratory or clinical signals	Introduce real-time reporting dashboards aligned with 7-1-7 targets
Community latency	Delayed reporting due to mistrust, stigma, poor access or absent reporting channels	Transmission continues outside formal surveillance	Strengthen and resource community-based surveillance systems

## The five dimensions of surveillance latency

Biological latency refers to the period in which infected people have non-specific symptoms or do not yet appear clinically severe. Early Ebola disease may present with fever, weakness, headache, muscle pain, vomiting or diarrhoea, all of which overlap with common infections in East Africa (4, 5). Clinical latency occurs when patients enter the health system but are not immediately recognised as possible Ebola cases. This is not necessarily poor clinical practice. In malaria-endemic settings, a febrile patient is statistically more likely to have malaria or another common infection than Ebola. The vulnerability is structural: rare but high-consequence infections can hide behind common symptoms, and the diagnostic infrastructure needed to distinguish them is often absent at the point of first contact. Diagnostic latency reflects delays in specimen collection, safe transport, laboratory processing and result communication. Rapid and reliable testing is central to Ebola control, but access is often constrained by biosafety requirements, laboratory distribution and field logistics (13). Administrative latency describes the delay between a suspected or confirmed signal and organised public health action. A result must move through facility, district, national and sometimes international reporting systems before contact tracing, isolation, risk communication and cross-border alerts begin. Each step can add time, and each depends on digital and communication infrastructure that may be unavailable. Community latency occurs when illness is recognised locally but not reported through formal systems. Fear, stigma, mistrust, poor transport, livelihood concerns and previous negative experiences with outbreak response can delay care-seeking or notification, as can the simple absence of a trusted channel through which to report. In such settings, communities may know that something unusual is happening before the health system does. The conceptual framework proposed in this article is illustrated in Figure 1, which highlights the cumulative delays that contribute to surveillance latency and identifies key points where intervention may shorten the interval between disease emergence and public health response.

**Figure 1:**
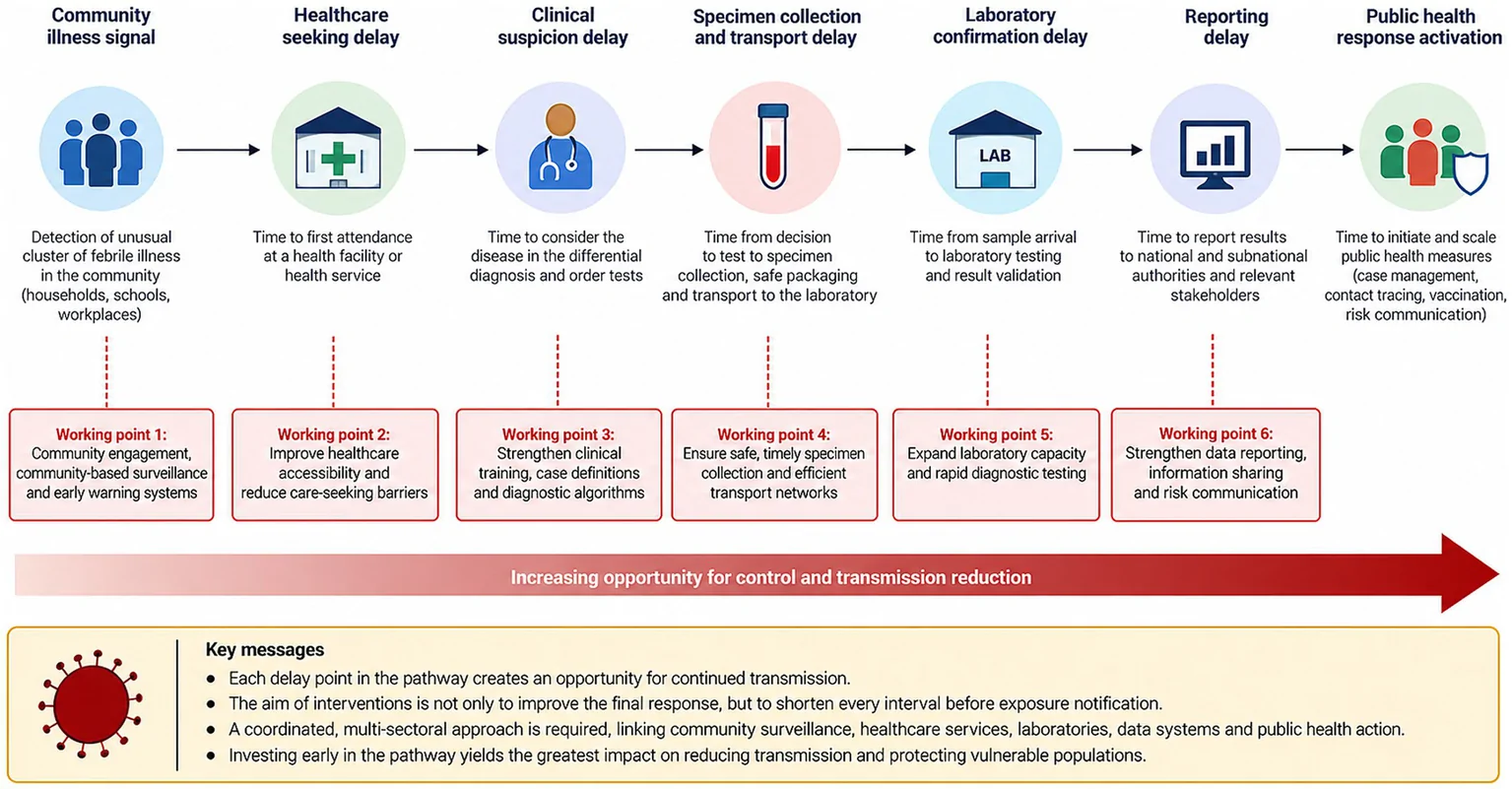
Conceptual pathway of surveillance latency in Ebola detection.

The figure illustrates sequential delays occurring between the initial community recognition of illness and activation of a public health response. Each delay represents a potential opportunity for continued transmission. The framework emphasises that preparedness should focus not only on outbreak response capacity but also on reducing latency throughout the detection pathway.

## Evidence of detection delay from recent outbreaks

The argument is concrete when set against documented delays. In the 2022 Uganda Sudan virus disease outbreak, the first case was confirmed on 20 September 2022, but retrospective investigation traced symptom onsets back to 8 August 2022 and identified community deaths with Ebola-like illness as far back as August; detection was delayed by approximately 6 weeks (1, 15). During that silent interval, transmission continued: symptom onsets eventually ranged from 8 August to 27 November 2022, the outbreak produced 142 confirmed and 22 probable cases, and 47% of patients died (15, 16). Much of the early delay was infrastructural and relational rather than a simple failure to act: most early case-patients, 26 (60%) of 43, had first sought care at private health facilities that were poorly integrated into the national surveillance system, and evaluations of affected sub-counties found systemic and knowledge gaps in integrated disease surveillance and response at both public and private facilities (17).

The same pattern recurs across the region and beyond. In the 2014–2016 West African epidemic, the index case is now thought to have died on 28 December 2013, yet the outbreak smouldered undetected for more than 3 months before the World Health Organization was notified on 23 March 2014, by which time transmission had crossed into Liberia and Sierra Leone (18, 19). Modelling of the 2022 Uganda outbreak underscores what such delay can cost: a scenario assuming a 5-month delay in mounting the response was estimated to produce many times the observed case burden, approaching the scale of the West African emergency (20). These figures are illustrative rather than exhaustive, but they show that the latency interval is measurable and that its length is consequential. Turnaround time for testing, days between symptom onset and care-seeking, and days between a laboratory signal and response activation are precisely the quantities a surveillance latency index would capture.

Table 2 draws on outbreak investigation reports and World Health Organization Disease Outbreak News to compare the interval between presumptive spillover or index-case onset and formal outbreak declaration with the eventual size of four recent filovirus disease outbreaks (1, 15–17, 21–26). Detection latency is approximated rather than precisely reconstructed, since none of the four events has a published, fully reconstructed transmission chain; the case counts reported at or near declaration are therefore a partial, lower-bound indication of transmission already under way rather than a complete enumeration. With this caveat, the pattern across these four events is consistent with the argument advanced in this Perspective: the shortest detection intervals, in Rwanda in 2024 and the Democratic Republic of the Congo in 2025, preceded the two smallest outbreaks, while the longest interval, in West Africa in 2013–2016, preceded by far the largest. Uganda’s 2022 outbreak, with an intermediate latency of approximately 6 weeks, produced an intermediate outbreak size. Case fatality ratios did not follow the same gradient, most plausibly because they are shaped by additional factors such as treatment-seeking behaviour, nosocomial transmission and access to supportive care rather than by detection speed alone. Four outbreaks are too few to establish a statistical relationship, and this comparison should be read as illustrative rather than confirmatory; a systematic analysis across a larger number of filovirus outbreaks, ideally using reconstructed transmission chains rather than reported case counts, would be needed to test the relationship formally. The comparatively short latency in the 2025 Bulape outbreak coincided with the use of an existing national DHIS2-based surveillance platform, discussed further below, that had already been configured for case-based Ebola and zoonotic disease reporting in the Democratic Republic of the Congo (21, 27).

**Table 2:** Detection latency and final outbreak size in four recent filovirus disease outbreaks.

Outbreak	Presumptive index case/spillover	Outbreak declared	Estimated detection latency	Cases reported at or near declaration	Final outbreak size and case fatality
Uganda, Sudan virus disease (2022)	Symptom onset retrospectively traced to approximately 8 August 2022	20 September 2022	~43 days (approx. 6 weeks)	Not systematically enumerated; retrospective review identified at least 43 early case-patients who had already sought care before recognition	164 cases (142 confirmed, 22 probable); 77 deaths (case fatality ratio 47%)
West Africa (Guinea-origin), Ebola virus disease (2013–2016)	Index case died 28 December 2013	WHO notified 23 March 2014	~85 days (approx. 12 weeks)	49 cases, 29 deaths (case fatality ratio 59%) reported at the time of WHO notification	28,616 cases; 11,310 deaths (case fatality ratio approx. 40%)
Rwanda, Marburg virus disease (2024)	Index case symptom onset retrospectively traced to approximately 8 September 2024	27 September 2024	~19 days (approx. 2.7 weeks)	Not systematically reported before declaration	66 cases; 15 deaths (case fatality ratio 23%)
Democratic Republic of the Congo (Bulape), Ebola virus disease (2025)	Index case symptom onset 10 August 2025; presentation to hospital 20 August 2025	4 September 2025	25 days (approx. 3.6 weeks)	28 suspected cases, 15 deaths (case fatality ratio 54%) reported at declaration	64 cases (53 confirmed, 11 probable); 45 deaths (case fatality ratio 70%)

## Why East Africa remains vulnerable

Several features make surveillance latency especially important in East Africa. The region contains busy cross-border corridors linking Uganda, the Democratic Republic of the Congo, South Sudan, Rwanda, Kenya and Tanzania. Formal borders do not always reflect daily patterns of trade, family life, displacement, work or care-seeking. Ecological risk also persists. Fruit bats and other wildlife interfaces remain relevant to filovirus spillover, while land-use change, hunting, mining, displacement and forest-edge settlement may increase human contact with reservoir or intermediate hosts (28, 29). Health-system capacity is uneven. National reference laboratories and emergency operations centres may be strong, yet peripheral facilities may lack reliable transport, electricity, personal protective equipment, digital reporting and diagnostic access; this uneven distribution of infrastructure is the material substrate of the geography of waiting. The 2022 Uganda Sudan virus outbreak illustrates both regional capability and continuing vulnerability; Uganda declared the outbreak on 20 September 2022, and subsequent reports documented 142 confirmed and 22 probable cases (1, 16). Community trust is also decisive. During the 2018–2020 DRC Ebola outbreak, surveillance and response occurred in areas affected by insecurity and mistrust, making community engagement essential rather than optional (2, 11). Where people fear isolation, stigma or loss of livelihood, or where no trusted reporting channel exists, they may delay reporting illness or avoid formal care.

## From response capacity to detection capacity

The dominant preparedness model asks whether countries can respond quickly after an outbreak is declared. That question remains important, but it is incomplete. East Africa also needs metrics that show how quickly a signal becomes visible. Three indicators should be measured routinely: median time from symptom onset to healthcare presentation, median time from healthcare presentation to laboratory confirmation, and median time from laboratory confirmation to public health action. Together, these indicators could form a surveillance latency index. Such an index would complement, not replace, the 7-1-7 target: 7-1-7 records whether timeliness benchmarks were met, while a latency index would decompose the interval and show where, geographically and infrastructurally, the time accumulates (8).

Such an index would show where delays accumulate. A district may have trained staff but poor transport. Another may have transport but weak community reporting. A third may collect specimens quickly but wait too long for laboratory confirmation. Read through an infrastructural lens, each pattern points to a specific missing resource rather than to negligence. The aim is not blame, but targeted correction.

## A regional framework for reducing surveillance latency

Real-time syndromic surveillance should be strengthened in high-risk districts. Facilities should be able to report unusual clusters of fever, unexplained deaths, haemorrhagic symptoms and healthcare-worker illness rapidly. Event-based surveillance should complement indicator-based systems. Informal signals from community health workers, religious leaders, schools, transport workers, traditional healers and local radio can reveal unusual events before routine systems detect them (9, 10, 12). Community health workers should be treated as early-warning infrastructure. Their role should extend beyond health education. They should be trained, paid, protected and connected to escalation pathways that move unusual illness signals to district authorities within hours rather than weeks. Decentralised molecular diagnostics should also be expanded. Not every district can host a high-containment laboratory immediately, but regional networks, mobile laboratories, safe specimen transport and validated rapid-testing pathways can shorten diagnostic latency (13).

Digital case-based surveillance platforms can accelerate several of these steps simultaneously. In the Democratic Republic of the Congo, the national District Health Information Software 2 (DHIS2) platform, already used for routine health information management and integrated disease surveillance, was configured with a dedicated Ebola virus disease tracker during the 2025 Bulape outbreak, allowing suspected cases, contacts, laboratory results and vaccination data to be entered from mobile devices in near real time and shared across facility, provincial and national levels (21). The same national DHIS2 infrastructure has separately been extended to animal health and zoonotic disease surveillance in Kongo Central province, integrating laboratory, vaccination and bite-case data for priority zoonoses, including Ebola virus disease, within a One Health framework (27). These applications illustrate how an existing, locally owned health information system can be adapted rapidly for outbreak-specific case-based surveillance rather than requiring a new platform to be built during an emergency, shortening both the administrative and diagnostic components of surveillance latency summarised in Table 1. Systems such as DHIS2 do not by themselves resolve community or clinical latency, but they can compress the interval between a laboratory or facility signal and its visibility to decision-makers, and their existing deployment across much of East and Central Africa makes them a practical foundation for the real-time reporting dashboards recommended above.

Cross-border surveillance agreements should be strengthened. Border closures alone may push movement into informal crossings. A more realistic approach is coordinated risk communication, shared alerts, compatible case definitions and rapid data exchange between neighbouring countries. Across all of these measures, the orientation is the same: to build the physical, technical and relational infrastructure whose absence produces waiting, and to align the resulting gains with measurable targets such as 7-1-7.

## Discussion

Ebola preparedness should be judged by speed of detection as much as speed of response. East Africa has made meaningful progress in outbreak response, clinical care, vaccination strategy and public health coordination. Yet the interval before recognition remains a dangerous blind spot. The geography of waiting helps explain why outbreaks unfold differently across locations. The same virus may produce different epidemic trajectories depending on road access, laboratory distance, reporting culture, community trust and cross-border movement. Latency is therefore not merely a technical delay. It is a social, geographical and infrastructural condition.

The framework also appears applicable beyond Ebola virus disease, and the same infrastructural delays are visible in recent events. During Rwanda’s first Marburg virus disease outbreak in 2024, epidemiological analysis traced the index case to around 8 September, nearly 3 weeks before the outbreak was declared on 27 September; because Marburg had never previously been recorded in the country, initial diagnosis was delayed, and most cases and deaths occurred in healthcare workers in the first 2 weeks after declaration (22, 24). The pattern mirrors Ebola: a rare pathogen presenting with non-specific symptoms, absent prior experience, and a detection interval during which transmission concentrated in clinical settings. Cholera offers a contrasting but related example. Where surveillance and laboratory confirmation are weak, cholera is frequently recognised only after community transmission is established; evaluations at Ugandan points of entry have used 7-1-7 timeliness metrics precisely because detection and notification of cholera can lag emergence (30). In each case the geography of waiting explains the latency in the same terms: detection is slow where the physical, technical and relational infrastructure for early recognition is missing, and fast where it is present. Surveillance latency may therefore represent a broader health-security indicator capable of informing preparedness planning across multiple epidemic-prone diseases.

A further caveat concerns the economic case for reducing surveillance latency. The comparison in Table 2 is consistent with the intuitive claim that faster detection limits eventual outbreak size, and by extension the cost of response, treatment and lost productivity. However, this Perspective has not conducted, and is not aware of, a formal economic evaluation that quantifies the cost per case or per death averted by specific investments in syndromic surveillance, decentralised diagnostics or community reporting systems for Ebola preparedness in East Africa. Claims about cost-effectiveness should therefore be treated as a plausible hypothesis grounded in the observed relationship between latency and outbreak size, rather than as an established economic finding. Prospective costing studies that link specific surveillance investments to downstream case numbers, treatment costs and productivity losses, ideally using the latency index proposed above as an explanatory variable, would be needed to substantiate or refine this claim.

This framework also avoids an unrealistic search for perfect surveillance. No system will detect every spillover immediately. The practical goal is to reduce waiting time enough that outbreaks are found while they are still small, traceable and locally containable.

## Conclusion

East Africa’s permanent fight against Ebola will not be won only through emergency treatment centres, vaccines or international response teams. Those tools matter, but they arrive too late if transmission has already expanded silently. Surveillance latency should become a central preparedness indicator. The geography of waiting framework shows how delays in symptom recognition, clinical suspicion, testing, reporting and response create opportunities for Ebola transmission before formal outbreak recognition, and it locates the cause of those delays in the uneven distribution of physical, technical and relational infrastructure. Reducing them requires investment in community-based surveillance, event-based surveillance, decentralised diagnostics, cross-border coordination and trusted communication, aligned with performance targets such as 7-1-7. Preparedness should ultimately be measured not only by how rapidly outbreaks are controlled after detection, but by how little time the virus is allowed to move before it is seen.

## Data Availability

The original contributions presented in the study are included in the article/supplementary material, further inquiries can be directed to the corresponding author.
